# Enhanced Dielectric Properties and Antibacterial Activity of Natural Rubber by Modification with Poly(Acrylic Acid-Co-Acrylamide) Incorporating Silver Nanoparticles and Titanium Dioxide

**DOI:** 10.3390/polym16223218

**Published:** 2024-11-20

**Authors:** Supharat Inphonlek, Supawat Kotchapradit, Boonruang Marungsri, Yupaporn Ruksakulpiwat, Chaiwat Ruksakulpiwat

**Affiliations:** 1 School of Polymer Engineering, Institute of Engineering, Suranaree University of Technology, Nakhon Ratchasima 30000, Thailand; supharat.inph@gmail.com; 2 Research Center for Biocomposite Materials for Medical Industry and Agricultural and Food Industry, Suranaree University of Technology, Nakhon Ratchasima 30000, Thailand; 3 School of Electronic Engineering, Institute of Engineering, Suranaree University of Technology, Nakhon Ratchasima 30000, Thailand; supawat@sut.ac.th; 4 School of Electrical Engineering, Institute of Engineering, Suranaree University of Technology, Nakhon Ratchasima 30000, Thailand; bmshvee@sut.ac.th

**Keywords:** natural rubber composites, silver nanoparticles, titanium dioxide, dielectric properties, antibacterial activity

## Abstract

This work aims to enhance natural rubber’s dielectric properties and antibacterial activity by incorporating silver nanoparticles and titanium dioxide. Deproteinized natural rubber (DPNR) was modified through the graft copolymerization of acrylic acid and acrylamide using N′, N′-Methylenebisacrylamide as a crosslinking agent, resulting in poly(acrylic acid-co-acrylamide)-modified, deproteinized natural rubber (MDPNR). This modification facilitated coordination with silver ions and interaction with titanium dioxide. Silver nanoparticles were generated under heat and pressure. Modified natural rubber composites containing silver nanoparticles and titanium dioxide (MDPNR/Ag-TiO_2_) were prepared. Scanning electron microscopy (SEM) revealed well-distributed silver in the modified natural rubber matrix, while agglomeration of titanium dioxide was observed at a high loading. Both MDPNR and MDPNR/Ag-TiO_2_ showed high thermal stability compared to DPNR. The MDPNR/Ag-TiO_2_ composites exhibited higher Tg and lower tan δ, indicating higher stiffness due to the restriction of chain movement compared to that in MDPNR. DPNR exhibited a low dielectric constant, enhanced by poly(acrylic acid-co-acrylamide) modification and silver nanoparticle/titanium dioxide incorporation. Incorporating 0.5 phr of AgNO_3_ and 2.5 phr of TiO_2_ in the composites increased the dielectric constant by 1.33 times compared to that of MDPNR. MDPNR showed no antibacterial activity, while the MDPNR/Ag-TiO_2_ composites exhibited promising antibacterial activity against *Staphylococcus aureus* and *Escherichia coli*.

## 1. Introduction

Composite materials with dielectric properties have garnered interest across various applications, including energy storage systems, flexible electronic equipment, sensors, and electrical robots [1,2,3]. A high dielectric constant allows for greater electrical energy storage, making materials more efficient for power storage systems. The development of energy storage devices with antibacterial properties represents a novel concept, integrating both energy storage capabilities and multifunctionality. Such systems can be highly beneficial for applications, such as healthcare storage devices and wearable electronics. Devices in healthcare are often exposed to bacteria, including *Staphylococcus aureus* (*S. aureus*) and *Escherichia coli* (*E. coli*). *S. aureus* is a Gram-positive bacterium, while *E. coli* is a Gram-negative bacterium. These bacteria are known to cause infections, particularly in vulnerable patients. Devices with antibacterial properties are safer because they reduce the risk of bacterial contamination that may come into contact with the skin or human body. Moreover, antibacterial materials prevent the growth of bacteria that can degrade the materials over time, thereby prolonging the shelf life of devices. Therefore, these devices require materials with biocompatibility, environmental friendliness, good thermal and mechanical stability, an enhanced dielectric constant, and antibacterial properties.

Among the polymers, natural rubber shows promise as a biopolymer matrix for preparing composite materials [4,5,6]. Natural rubber, derived from the rubber tree (*Hevea Brasiliensis*), exhibits high elasticity, flexibility, and tensile strength. It is easily processed and compounded with other materials, and is capable of forming films [7,8]. Moreover, it is biodegradable and renewable, making it more environmentally friendly than synthetic alternatives [9]. Considering these properties, natural rubber is an attractive choice for diverse applications. However, natural rubber has certain limitations, such as a low dielectric constant and susceptibility to bacterial contamination, which restrict its use in advanced applications like electronics, energy storage devices, healthcare, and environmental systems [10]. Enhancing the functional properties of natural rubber to meet these demands is an area of growing interest. One approach involves incorporating functional fillers, such as graphene and titanium dioxide [11,12,13]. To improve the compatibility of natural rubber with polar fillers, modified natural rubber composites have been developed. Previous work has demonstrated that graft the copolymerization of polar monomers, such as acrylic acid and acrylamide, onto deproteinized natural rubber can enhance its polarity and interactions with fillers [14]. This environmentally friendly modification method utilizes water as a medium, avoiding harmful organic solvents. The process involves using deproteinized natural rubber (DPNR), which suppresses side reactions during chemical modification, enhances biocompatibility, and reduces allergic reactions, making it particularly suitable for medical and sensitive applications [15]. Modified DPNR/filler composites exhibit improved mechanical and thermal properties. Furthermore, poly(acrylic acid-co-acrylamide) is capable of coordination with silver ions and interaction with titanium dioxide. Composites of poly(acrylic acid-co-acrylamide)-modified DPNR incorporating silver nanoparticles and titanium dioxide show enhanced compressive moduli, good structural stability, and effectiveness in dye removal [16]. Thus, the modification of deproteinized natural rubber by introducing polar functional components can enhance its compatibility with fillers, potentially improving the properties of modified natural rubber composites for a variety of applications.

Titanium dioxide (TiO_2_), known for its favorable physical and chemical properties, including electronic properties, photocatalytic activity, chemical stability, and low toxicity, is particularly interesting [17,18]. Bunriw et al. prepared natural rubber/TiO_2_ composites as environmentally friendly materials for triboelectric nanogenerator (TENG) applications, aiming to harvest mechanical energy into electricity [19]. The addition of TiO_2_ improved the dielectric constant of the natural rubber composites. The enhancement of the TENG electrical output with the highest power density of 237 mW/m^2^ was achieved. Therefore, these natural rubber/TiO_2_ composites are promising for developing large-scale energy harvesting devices.

Silver-containing materials exhibit photocatalytic activity and antibacterial properties [20,21,22,23]. Silver nanoparticles may penetrate bacteria cells and release silver ions that disrupt metabolic processes and inhibit cell growth. Appamato et al. developed a triboelectric nanogenerator using a natural rubber–silver nanocomposite applied as a shoe insole to harvest human footstep energy [24]. These Ag nanoparticles created interfacial polarization between the conductive metal nanoparticle and the insulating polymer, thereby improving the dielectric constant and electrical output. Additionally, they exhibited antibacterial activity against *Staphylococcus aureus*, the bacterium responsible for foot odor. Zheyan Soo et al. prepared silver-doped TiO_2_ nanofibers for antibacterial applications [25]. The silver-doped TiO_2_ nanofibers demonstrated stronger antibacterial activity than bare TiO_2_ nanofibers, indicating a synergistic enhancement in antibacterial performance through the combination of Ag nanoparticles and TiO_2_. Among the nanofibers, those with 2% Ag content showed the highest antibacterial performance, reducing *Salmonella* Albany and *S. aureus* by 5.92 ± 0.00 and 1.38 ± 0.07 logCFU, respectively.

This study focuses on the preparation of poly(acrylic acid-co-acrylamide)-modified, deproteinized natural rubber composites incorporating silver nanoparticles and titanium dioxide (MDPNR/Ag-TiO_2_), aiming to enhance the dielectric constant and antibacterial activity of natural rubber. Incorporating silver nanoparticles and titanium dioxide into the modified natural rubber matrix has emerged as a promising strategy for imparting additional functionalities. This work contributes to the development of modified natural rubber composites for advanced applications, such as energy storage devices in healthcare, where high dielectric constants are essential for effective energy storage and transfer. The biocompatibility, flexibility, and antibacterial properties of modified natural composites further enhance their potential for use in flexible and wearable medical devices. This research evaluates the physical, thermal, and dielectric properties of the composites, as well as their antibacterial performance against *Staphylococcus aureus* and *Escherichia coli.*

## 2. Materials and Methods

### 2.1. Materials

Natural rubber (NR), with a 60% dry rubber content and preserved using high ammonia, was received from Chemical and Materials Co., Ltd. (Bangkok, Thailand). Urea was purchased from RCI Labscan Limited (Bangkok, Thailand). Sodium dodecyl sulfate (SDS) and acrylamide (AM) monomer were acquired from Loba Chemie Pvt. Ltd. (Mumbai, India). Acrylic acid (AA) monomer and cumene hydroperoxide (CHP) initiator were supplied by Aldrich (St. Louis, MO, USA). The AA monomer was passed through a column packed with alumina adsorbent for purification. Tetraethylene pentamine (TEPA) was purchased from Acros Organics (Geel, Belgium), and Terric16A (10 wt%) was obtained from the Rubber Authority of Thailand (Bangkok, Thailand). N′, N′-Methylenebisacrylamide (MBA) was provided by Alfa Aesar (Ward Hill, MA, USA). Silver nitrate (AgNO_3_) was obtained from Quality Reagent Chemical, controlled by QReC New Zealand (Rawang, Malaysia). Titanium dioxide (TiO_2_), the anatase phase with an oil absorption value of 26 g/100 g and a residue from a 45 µm sieve of ≤0.1%, was obtained from Cernic International Co., Ltd. (Nakhon Pathom, Thailand). Throughout the study, deionized (DI) water was used.

### 2.2. Preparation of Poly(Acrylic Acid-Co-Acrylamide)-Modified, Deproteinized Natural Rubber Comprising Silver Nanoparticles and Titanium Dioxide (MDPNR/Ag-TiO_2_) Composites

**Figure 1:**
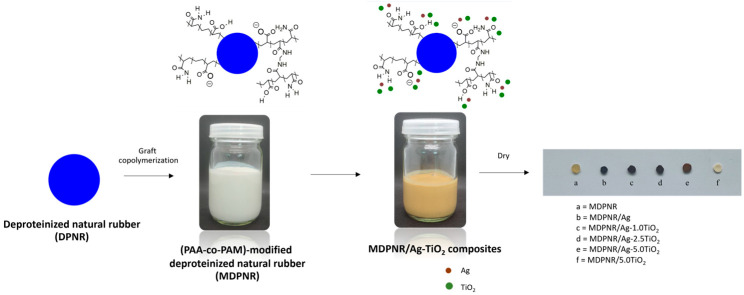
Schematic illustration of the preparation of the MDPNR/Ag-TiO_2_ composites and the visual appearance of the obtained samples after drying.

The poly(acrylic acid-co-acrylamide)-modified, deproteinized natural rubber composites containing silver nanoparticles and titanium dioxide (MDPNR/Ag-TiO_2_) were prepared following the method outlined by Inphonlek et al. [16]. First, deproteinized natural rubber (DPNR) was obtained by removing proteins from NR latex, as described by Kawahara et al. [26]. The NR latex was mixed with 0.1%w urea and 1%w SDS under continuous magnetic stirring for 60 min. The mixture was then centrifuged at 15,000 rpm for 30 min to separate the rubber phase from the aqueous phase containing proteins and impurities. The washing process was repeated twice by redispersing the cream fraction in 1%w SDS, followed by centrifugation. The rubber phase was collected and dispersed in water in the presence of Terric16A as a stabilizer. Next, the poly(acrylic acid-co-acrylamide)-modified, deproteinized natural rubber (MDPNR) was synthesized via graft copolymerization of acrylic acid and acrylamide, using MBA as the crosslinking agent. Briefly, DPNR latex was transferred to a reactor and stirred mechanically at 100 rpm under a nitrogen atmosphere. The following chemicals were added in sequence: CHP, acrylic acid (50 mol% neutralized with 20%w NaOH solution), acrylamide, MBA, and TEPA. In this study, the monomer content was set at 20 phr, maintaining a 70:30 weight ratio of acrylic acid to acrylamide. MBA was used at 1.00%w of the monomer. The CHP and TEPA contents were fixed at 1 phr. Polymerization was carried out for 6 h at 50 °C under a nitrogen atmosphere. Subsequently, the resulting MDPNR latex was mixed with a 1% *w*/*v* AgNO_3_ solution and a 10% *w*/*v* TiO_2_ dispersion. The composites were prepared with a fixed AgNO_3_ concentration of 0.5 phr and varying TiO_2_ contents of 1.0, 2.5, and 5.0 phr, denoted as MDPNR/Ag-1.0TiO_2_, MDPNR/Ag-2.5TiO_2_ and MDPNR/Ag-5.0TiO_2_, respectively. The mixture was stirred at 600 rpm under dark conditions for 30 min and then subjected to an autoclave (HVA-110, Hirayama Manufacturing Corporation, Saitama, Japan) at 120 °C and 15 psi for 50 min. The resulting product was dried in a hot-air oven at 60 °C for 24 h. Additionally, samples containing either AgNO_3_ or TiO_2_ alone, referred to as MDPNR/Ag and MDPNR/5.0TiO_2_ composites, respectively, were prepared for comparison. The prepared samples were stored for analysis, as shown in Figure 1.

### 2.3. Fourier Transform Infrared Spectroscopy

Attenuated total reflectance Fourier transform infrared spectroscopy (ATR-FTIR) was conducted to analyze the chemical structure of the prepared composites using an FTIR spectrophotometer (Tensor 27, Bruker, Billerica, MA, USA). Each spectrum was scanned from 4000 to 500 cm^−1^, with a resolution of 4 cm^−1^, and 64 scans were performed.

### 2.4. Morphological Analysis

The morphology of the prepared composites was observed using a scanning electron microscope (SEM). The dried samples were fixed on the stub and coated with gold under vacuum using a sputter coater (EM ACE600, Leica microsystems, Wetzlar, Germany). The surface morphology of samples was investigated using a JSM-7800F field emission scanning electron microscope (JEOL Ltd., Tokyo, Japan). Additionally, the element composition and distribution of the composites were determined by energy-dispersive spectroscopy coupled with SEM (SEM/EDS).

### 2.5. Thermogravimetric Analysis

The thermal stability of various types of composites was assessed using a thermogravimetric analyzer (TG 209 F3 Tarsus, Netzsch, Germany). For each sample, 10 mg of dried material was placed in a sample pan and heated from 50 to 600 °C at a heating rate of 10 °C/min. The measurement was carried out under a nitrogen atmosphere. The change in the remaining sample weight was continuously monitored throughout the measurement.

### 2.6. Dynamic Mechanical Analysis

The thermomechanical properties of the composites were evaluated using dynamic mechanical analysis (DMA) performed on a DMA850 instrument (TA Instruments, New Castle, DE, USA). A temperature sweep test was measured with a dynamic strain of 0.1% and a frequency of 1 Hz. The samples were tested over a temperature range from −80 to 100 °C, with a heating rate of 2 °C/min.

### 2.7. Dielectric Constant Testing

The dielectric constant was measured by using an impedance analyzer (Keysight E4294A, Agilent, Santa Clara, CA, USA) at room temperature. The samples were placed between the electrodes. The measurements were performed at varying frequencies ranging from 10^2^ to 10^7^ Hz.

### 2.8. Antibacterial Activity Evaluation

The antibacterial activity of the prepared composites was assessed using the disk diffusion method. *Staphylococcus aureus* TISTR 746 (*S. aureus*) and *Escherichia coli* TISTR 527 (*E. coli*) were chosen as representative Gram-positive and Gram-negative bacterial strains, respectively. The suspensions of microorganisms in CriterionTM Nutrient Broth (NB) were spread as thin layers on CriterionTM Mueller–Hinton (MH) agar in Petri dishes. Specimens measuring 6 mm in diameter were UV-sterilized for 1 h on each side prior to testing. Subsequently, the specimens were placed on top of the smeared agar, and then the plate was incubated at 37 °C for 24 h. Amoxicillin antibiotic disks were used as the positive control. The absence of bacterial growth, indicated by clear inhibition zones around the disk specimens, signified that those inhibitory concentrations had been achieved.

## 3. Results

### 3.1. FTIR Analysis

Modified natural rubber composites incorporating silver nanoparticles and titanium dioxide were prepared in this study. The deproteinized natural rubber was first modified by grafting with poly(acrylic acid-co-acrylamide) via emulsion graft copolymerization, acting as the polymeric matrix. The modified natural rubber was capable of forming coordination bonds with silver ions due to the presence of the carbonyl groups, carboxylate ions, and nitrogen atoms of amide groups in the polymeric matrix [27,28]. The silver ions were reduced to metallic silver atoms under a high temperature and applied pressure. These metallic silver atoms aggregate, forming clusters that grow into stable silver nanoparticles. Meanwhile, titanium dioxide can interact with modified natural rubber through hydrogen bonding [29]. As a result, the silver nanoparticles and titanium dioxide were distributed in the modified natural-rubber-based matrix.

**Figure 2:**
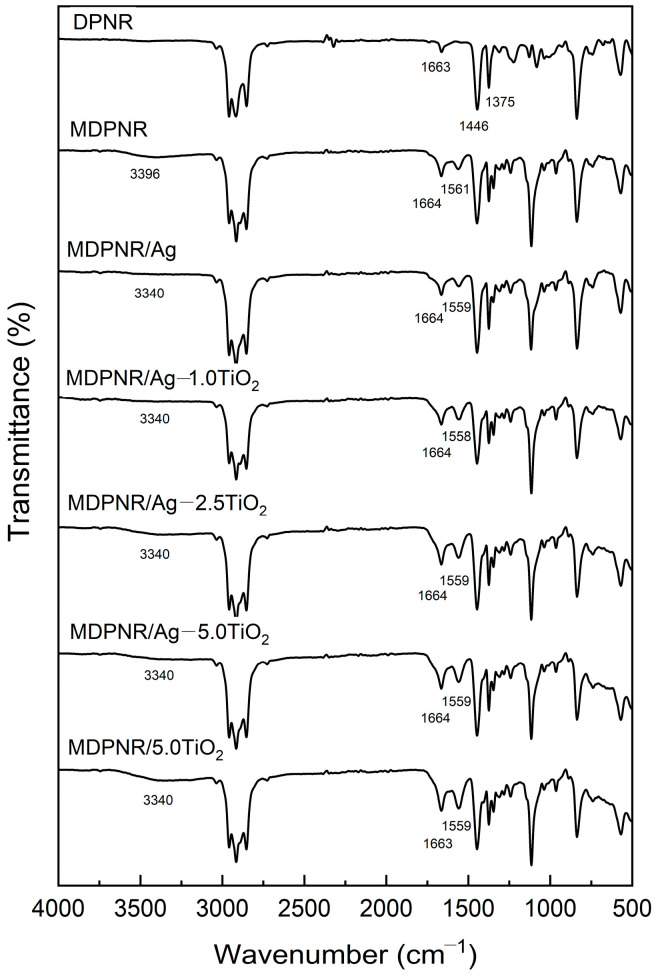
FTIR spectra of the DPNR, MDPNR, MDPNR/Ag, MDPNR/Ag-TiO_2_ with various TiO_2_ contents, and MDPNR/TiO_2_ composites.

The chemical functional groups in the samples were determined using FTIR analysis, as seen in Figure 2. In the FTIR spectrum of DPNR, peaks around 3000–2800 cm^−1^ were observed, attributed to the C-H stretching vibration of the polyisoprene backbone. Additionally, a peak at 1663 cm^−1^ was attributed to C=C stretching. Peaks at 1446 and 1375 cm^−1^ were observed, corresponding to -CH_2_ and -CH_3_ stretching vibration, respectively [30]. After modification through graft copolymerization, the FTIR spectrum of MDPNR exhibited a broad band around 3500–3000 cm^−1^, indicating the presence of O-H and the N-H stretching of poly(acrylic acid-co-acrylamide). A peak at 1664 cm^−1^ was attributed to the C=O stretching. Another peak at 1561 cm^−1^ corresponded to carboxylate, resulting from the partial neutralization of acrylic acid. The presence of new peaks confirmed the success of the modification process [14,31,32]. In the case of the MDPNR/Ag, MDPNR/Ag-TiO_2_, and MDPNR/TiO_2_ composites, their spectra exhibited characteristics similar to those of modified natural rubber. The C=O stretching vibrations remained unchanged at 1664 cm^−1^. However, the characteristic peaks of N-H stretching, O-H stretching, and carboxylate slightly shifted to a lower wavenumber. The peaks corresponding to N-H and O-H stretching vibrations shifted from 3396 to 3340 cm^−1^, and carboxylate peaks shifted from 1561 to 1559 cm^−1^. These corresponded to the results of the previous report by Anancharoenwong et al. [33]. These changes indicate the interactions of modified natural rubber with silver and titanium dioxide in the composites [34,35,36].

### 3.2. Morphology

**Figure 3:**
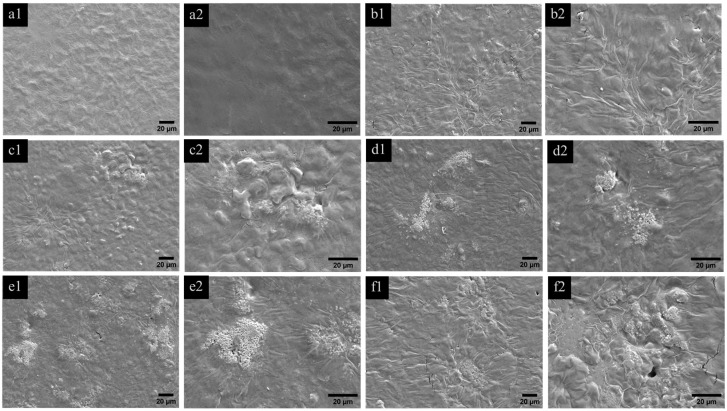
SEM images at ×500 and ×1000 magnifications of (**a1**,**a2**) MDPNR; (**b1**,**b2**) MDPNR/Ag; (**c1**,**c2**) MDPNR/Ag-1.0TiO_2_; (**d1**,**d2**) MDPNR/Ag-2.5TiO_2_; (**e1**,**e2**) MDPNR/Ag-5.0TiO_2_; (**f1**,**f2**) MDPNR/5.0TiO_2_.

**Figure 4:**
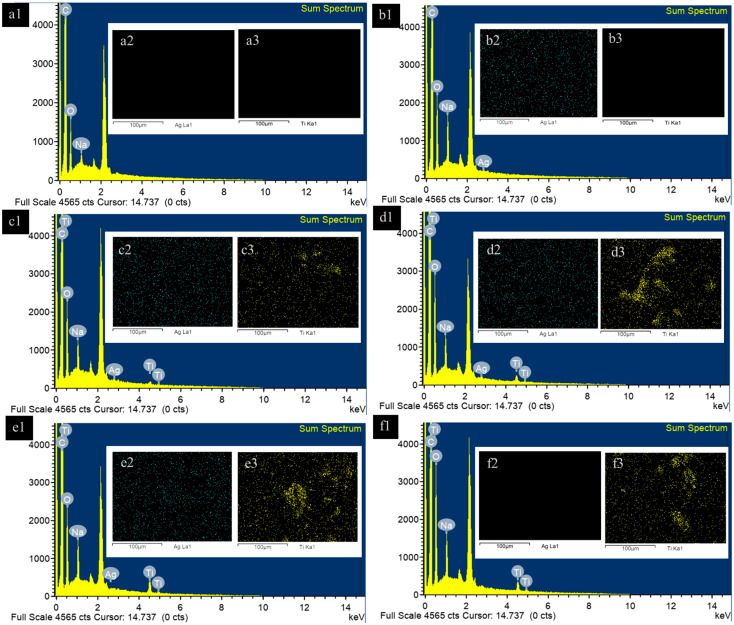
EDS spectra (first column) and EDS mappings, with green spots indicating silver (second column) and yellow spots indicating titanium (third column), of (**a1**–**a3**) MDPNR; (**b1**–**b3**) MDPNR/Ag; (**c1**–**c3**) MDPNR/Ag-1.0TiO_2_; (**d1**–**d3**) MDPNR/Ag-2.5TiO_2_; (**e1**–**e3**) MDPNR/Ag-5.0TiO_2_; (**f1**–**f3**) MDPNR/5.0TiO_2_.

The morphology of MDPNR and its composites, which include MDPNR/Ag, MDPNR/Ag-TiO_2_ with varying TiO_2_ contents, and MDPNR/TiO_2_, is illustrated in Figure 3. SEM images revealed that the surface of MDPNR appeared relatively smooth, whereas the composites exhibited rougher surfaces due to the distribution of solid particles within the natural-rubber-based matrix. At higher magnification, the titanium-dioxide-containing composites exhibited solid particles approximately 240 ± 61 nm in size, as measured using ImageJ software (IJ 1.46r image analyzer software). The elemental composition and distribution of the samples were investigated by EDS analysis. Figure 4 depicts the composites’ EDS spectra and mappings corresponding to Ag and Ti. The spectra revealed the presence of C, O, and Na in MDPNR. Additionally, a characteristic signal for Ag was observed in MDPNR/Ag [37], and signals corresponding to Ti appeared in MDPNR/5.0TiO_2_ [38,39]. The MDPNR/Ag-TiO_2_ composites with different TiO_2_ contents exhibited signals indicative of modified natural rubber, Ag, and Ti, confirming the incorporation of these elements in the composites. However, the Ag peak appeared faint, likely due to the relatively low Ag content in the composites. EDS mappings demonstrated that Ag was well-distributed on the sample surface. The modified natural rubber can act as a stabilizing agent for the formation of stable silver nanoparticles with good dispersibility within the polymer matrix. Ti was also dispersed in the modified natural-rubber-based matrix. However, titanium tended to agglomerate at a higher loading, particularly evident when incorporating 5 phr of titanium dioxide, with agglomerate sizes ranging from 0.82 to 12.28 µm. The elemental compositions of the composites from EDS analysis are summarized in Table 1. The results indicated that carbon was the dominant element in the composites, ranging from a 66.44 to 84.67 weight percentage and a 75.06 to 88.19 atomic percentage, due to its presence as the primary component of the modified natural rubber matrix. Incorporating silver nanoparticles and titanium dioxide into the composites reduced the weight and atomic percentages of carbon. The composites contained a small amount of silver, with values ranging a from 0.61 to 1.08 weight percentage and a 0.08 to 0.13 atomic percentage. An increase in the weight and atomic percentages of titanium was observed with higher titanium dioxide contents. The titanium weight and atomic percentages were increased from 1.53 to 8.21 and 0.42 to 2.36 for MDPNR/Ag-1.0TiO_2_, MDPNR/Ag-2.5TiO_2,_ and MDPNR/Ag-5.0TiO_2,_ respectively.

### 3.3. Thermal Properties

**Figure 5:**
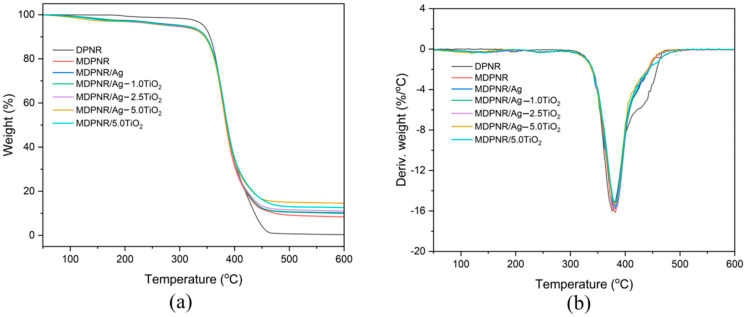
(**a**) TGA and (**b**) DTG thermograms of the DPNR, MDPNR, MDPNR/Ag, MDPNR/Ag-TiO_2_ composites with various TiO_2_ contents, and MDPNR/TiO_2_.

The thermal gravimetric analysis (TGA) and the first derivative of TGA (DTG) curves of the MDPNR/Ag-TiO_2_ composites, compared to those of DPNR and MDPNR, are presented in Figure 5. The TGA thermograms indicate weight loss occurring within the temperature range of 50 to 600 °C, followed by residue formation. Detailed parameters, such as the temperature at 5% weight loss (T_5_), temperature at maximum process rate (T_max_), and residue content, are provided in Table 2. As a result, DPNR decomposition occurred between 336 and 476 °C, and almost no residue remained at 600 °C. After modification of DPNR via grafting with poly(acrylic acid-co-acrylamide), MDPNR exhibited initial weight loss between 70 and 170 °C due to the evaporation of absorbed and bound water. Since the natural rubber was modified with a hydrophilic polymer, the MDPNR could absorb water molecules in its structure. Subsequent weight loss between 180 and 291 °C was attributed to the decomposition of carboxylic acid and amide side groups. Principal decomposition, involving the natural rubber and the polymer backbone of poly(acrylic acid-co-acrylamide), occurred at 336–476 °C [40,41]. The shift in T_max_ to a higher temperature than DPNR suggests the improved thermal stability of MDPNR. From the result, the decomposition characteristics of the MDPNR/Ag-TiO_2_ composites were similar to those of MDPNR. However, metallic silver and titanium dioxide in the composites reduced T_5_ compared to MDPNR. This may be due to the heat conductivity of silver and titanium dioxide, accelerating the decomposition process of side groups [42]. Nonetheless, the decomposition of the main components of the composites, comprising natural rubber modified with poly(acrylic acid-co-acrylamide), exhibited no difference in T_max_ values, indicative of the high thermal stability of the composites. Furthermore, the residues of the composites were higher than those of MDPNR, and they increased with increasing silver and titanium dioxide contents in the composites.

### 3.4. Dynamic Mechanical Properties

**Figure 6:**
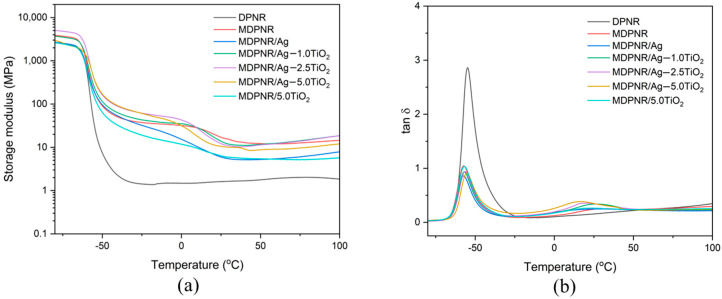
(**a**) Storage modulus and (**b**) loss tangent of composites as a function of temperature.

The viscoelastic behavior of the MDPNR/Ag-TiO_2_ composites was investigated through temperature sweep testing. Figure 6a,b illustrate the storage modulus (E′) and loss tangent (tan δ) of the composites over a temperature range from −80 to 100 °C, respectively. All samples displayed a high storage modulus in the glassy state at low temperatures, as shown in Figure 6a. With increasing temperature, the storage modulus gradually decreased due to the increased mobility of the polymer chains [43]. The storage modulus decreased significantly due to the transition to a rubbery state. In dynamic mechanical analysis, the material’s glass transition temperature (Tg) can be determined from the peak in the tan δ curve (Figure 6b). The viscoelastic properties are summarized in Table 3. As can be seen from the result, DPNR exhibited a single tan δ peak corresponding to the Tg of natural rubber, which was found to be −54.89 °C. The MDPNR showed two tan δ peaks. The first peak corresponded to the bulk of the modified natural rubber, while the second peak may be attributed to the polymeric chain network formed due to the modification. As observed in the bulk of the polymer, the Tg of MDPNR shifted to a lower temperature (−57.21 °C). This shift may be attributed to structural irregularities and intercalation between rubber chains, which decrease the number of chain entanglements and facilitate more effortless movement of the rubber chains. Interestingly, the Tg values of the MDPNR/Ag-TiO_2_ composites appeared to increase compared to MDPNR. The Tg values of MDPNR/Ag-TiO_2_ composites increased from −56.90 to −55.43 °C when TiO_2_ was increased from 1.0 to 5.0 phr. Simultaneously, the peak height of tan δ decreased, suggesting increased stiffness and the restriction of polymer chain movement, probably due to the interaction of silver and titanium dioxide with a modified natural rubber matrix [44]. Silver can coordinate with active functional group-containing modified natural rubber [45], while titanium dioxide can interact with modified natural rubber through polar–polar interaction and hydrogen bonding between the hydroxylated titanium dioxide surface and modified natural rubber [46]. These findings suggest that the composites exhibit good stability against deformation.

### 3.5. Dielectric Property

**Figure 7:**
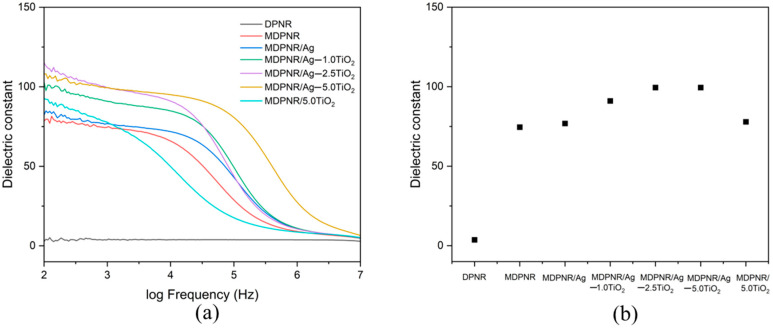
(**a**) Dielectric constant of the DPNR, MDPNR, and MDPNR/Ag-TiO_2_ composites with various TiO_2_ contents as a function of frequency and (**b**) dielectric constant of various types of composites at 1 kHz.

The materials’ dielectric properties are important in studying their electrical response in an external electric field. The dielectric constant measures the ability of materials to store electrical energy from an electric field in their structure. Figure 7a illustrates the dielectric constants of DPNR, MDPNR, and MDPNR/Ag-TiO_2_ composites across frequencies ranging from 10^2^ to 10^7^ Hz. DPNR exhibited a low dielectric constant due to the non-polar characteristic of natural rubber. The MDPNR and MDPNR/Ag-TiO_2_ composites demonstrated a high dielectric constant at a low frequency. However, the dielectric constant decreased with increasing frequency, possibly due to the molecular movement’s incapability and decreased orientation polarization at higher frequencies [47,48]. A comparison of dielectric constants of samples at 1 kHz is presented in Figure 7b. Notably, the dielectric constant of MDPNR was 74.54, which was higher than that of DPNR. This increase can be attributed to the modification of natural rubber with poly(acrylic acid-co-acrylamide), introducing a polar component to its structure [49,50,51].

Furthermore, introducing silver nanoparticles and titanium dioxide enhanced the dielectric constant, suggesting that the composites possess more incredible electrical energy [52]. Silver and titanium dioxide act as fillers dispersed in the natural rubber-based matrix. Interfacial polarization occurred within the composites due to differences in the polarizations of the matrix and fillers, increasing the dielectric constant [53]. Indeed, the dielectric constant increases with higher titanium dioxide contents. Specifically, the dielectric constant values of MDPNR/Ag-TiO_2_ composites increased to 91.03 and 99.46 when TiO_2_ was added at 1.0 and 2.5 phr, respectively. At 5.0 phr, the dielectric constant does not differ significantly from that observed at 2.5 phr due to the agglomeration of titanium dioxide at a high loading. However, it exhibited a high dielectric constant in the broad frequency range. These correspond to the report by Sintharm et al., describing that the dielectric behavior of composites depends on the dielectric property of the polymer matrix and filler, chemical composition, chemical structure, and filler dispersion in the composites [54]. Thus, the enhancement of the dielectric constant of natural rubber through modification with poly(acrylic acid-co-acrylamide), incorporating silver nanoparticles and titanium dioxide, improved the dielectric constant, and made the composites more efficient for applications.

### 3.6. Antibacterial Activity

**Figure 8:**
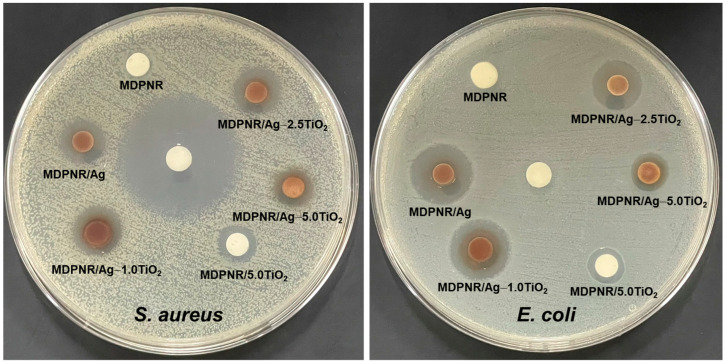
Disk diffusion test of MDPNR, MDPNR/Ag, MDPNR/Ag-TiO_2_ with various TiO_2_ contents, and MDPNR/TiO_2_ composites.

The antibacterial behavior of the MDPNR/Ag-TiO_2_ composites against *S. aureus* and *E. coli*, compared to that of MDPNR, was examined, as shown in Figure 8. The results indicated that clear zones were not observed around the MDPNR samples on the agar plates against *S. aureus* and *E. coli*, suggesting no antibacterial activity. Interestingly, all composites demonstrated the ability to inhibit bacterial growth and form clear zones around the samples for both *S. aureus* and *E. coli*. The size of the clear zones for the samples was summarized in Table 4. It was observed that the composites composed of silver nanoparticles and/or titanium dioxide showed the inhibition zone, suggesting the effectiveness of silver nanoparticles and titanium dioxide for antibacterial activity. The antibacterial activity may be attributed to the penetration of silver particles and the release of silver ions, which interfere with bacterial cell membranes, disrupting their functions and leading to cell death [55]. Additionally, titanium dioxide may penetrate bacterial cell walls and disrupt essential cellular functions. As a result, the size of the clear zone for MDPNR/Ag composites against *S. aureus* and *E. coli* was measured as 12.0 ± 1.0 mm and 15.3 ± 0.6 mm, respectively, showing intense antibacterial activity. For the MDPNR/Ag-TiO_2_ composites, the addition of titanium dioxide at 1.0 and 2.5 phr did not differ from the size of the clear zones compared to MDPNR/Ag. The inhibition zone was 11.7 ± 0.6, 12.0 ± 0.0 mm for *S. aureus* and 14.0 ± 0.0, 13.7 ± 0.6 mm for *E. coli* when titanium dioxide was added at 1.0 and 2.5 phr, respectively. However, upon increasing the titanium dioxide content to 5.0 phr, the inhibition zone against *S. aureus* and *E. coli* decreased to 10.7 ± 1.5 and 11.3 ± 2.5 mm, respectively. This reduction may have been due to the high titanium dioxide content, which could have resulted in poor distribution in the modified natural rubber matrix, as observed in SEM images, thereby reducing antibacterial efficacy. Nevertheless, the antibacterial efficiency of the MDPNR/Ag-5.0TiO_2_ composites remained more remarkable than that of MDPNR/5.0TiO_2_, which contains only titanium dioxide. It was indicated that the incorporation of silver nanoparticles and titanium dioxide promoted the antibacterial activity of natural rubber composites. Therefore, these MDPNR/Ag-TiO_2_ composites exhibited antibacterial activity for both *S. aureus* and *E. coli* and could have potential for many applications.

## 4. Conclusions

The modification of deproteinized natural rubber (DPNR) through graft copolymerization facilitated the introduction of functional groups and enhanced interaction with silver nanoparticles and titanium dioxide. This modification was successfully achieved in this work, resulting in poly(acrylic acid-co-acrylamide)-modified, deproteinized natural rubber incorporating silver nanoparticles and titanium dioxide (MDPNR/Ag-TiO_2_) composites. SEM-EDS mappings revealed that silver nanoparticles exhibited a uniform distribution in the modified natural rubber matrix. However, titanium dioxide tended to agglomerate at higher loadings, particularly noticeable at 5 phr of titanium dioxide. The MDPNR/Ag-TiO_2_ composites demonstrated good thermal stability. The shift in Tg to a higher temperature and the decrease in the tan δ peak height indicate increased stiffness and resistance to deformation in the MDPNR/Ag-TiO_2_ composites. The modification of DPNR with polar components, along with the presence of silver nanoparticles and titanium dioxide, improved the dielectric properties of the composites. Additionally, the existence of silver nanoparticles and titanium dioxide endowed the composites with antibacterial activity against *S. aureus* and *E. coli*. These composites, demonstrating good thermal stability, improved dielectric properties, and antibacterial activity, could have useful application in various fields.

## Figures and Tables

**Table 1:** Elemental composition derived from EDS results of the composites.

Samples	Weight (%)					Atomic (%)				
	C	O	Na	Ag	Ti	C	O	Na	Ag	Ti
MDPNR	84.67	14.57	0.76	0.00	0.00	88.19	11.39	0.41	0.00	0.00
MDPNR/Ag	75.88	19.40	3.65	1.08	0.00	82.06	15.75	2.06	0.13	0.00
MDPNR/Ag-1.0TiO_2_	75.63	19.65	2.23	0.96	1.53	82.17	16.03	1.26	0.12	0.42
MDPNR/Ag-2.5TiO_2_	70.93	19.92	1.72	0.75	6.69	80.11	16.89	1.01	0.09	1.90
MDPNR/Ag-5.0TiO_2_	69.26	19.69	2.22	0.61	8.21	79.31	16.93	1.33	0.08	2.36
MDPNR/5.0TiO_2_	66.44	25.73	2.93	0.00	4.90	75.06	21.82	1.73	0.00	1.39

**Table 2:** Thermal degradation temperature and residue from TGA curves of the DPNR, MDPNR, MDPNR/Ag, MDPNR/Ag-TiO_2_ composites with various TiO_2_ contents, and MDPNR/TiO_2_.

Samples	T_5_ (°C)	T_max_ (°C)	Residue at 600 °C (%)
DPNR	343.61	376.61	0.36
MDPNR	307.69	381.69	8.38
MDPNR/Ag	306.44	380.96	10.01
MDPNR/Ag-1.0TiO_2_	302.27	383.27	10.28
MDPNR/Ag-2.5TiO_2_	279.08	381.08	11.02
MDPNR/Ag-5.0TiO_2_	291.98	380.98	14.57
MDPNR/5.0TiO_2_	301.16	380.16	12.62

**Table 3:** Viscoelastic properties of MDPNR/Ag-TiO_2_ composites.

Samples	Tan δ Peak Position (°C)	Tan δ Peak Height
DPNR	−54.89	2.861
MDPNR	−57.21	1.045
MDPNR/Ag	−57.28	0.858
MDPNR/Ag-1.0TiO_2_	−56.90	0.933
MDPNR/Ag-2.5TiO_2_	−56.41	0.939
MDPNR/Ag-5.0TiO_2_	−55.43	0.908
MDPNR/5.0TiO_2_	−56.83	1.036

**Table 4:** Antibacterial inhibition zone of MDPNR, MDPNR/Ag, MDPNR/Ag-TiO_2_ with various TiO_2_ contents, and MDPNR/TiO_2_ composites.

Samples	Zone of Inhibition (mm)	
	*S. aureus*	*E. coli*
MDPNR	0.0 ± 0.0	0.0 ± 0.0
MDPNR/Ag	12.0 ± 1.0	15.3 ± 0.6
MDPNR/Ag-1.0TiO_2_	11.7 ± 0.6	14.0 ± 0.0
MDPNR/Ag-2.5TiO_2_	12.0 ± 0.0	13.7 ± 0.6
MDPNR/Ag-5.0TiO_2_	10.7 ± 1.5	11.3 ± 2.5
MDPNR/5.0TiO_2_	9.3 ± 1.2	8.7 ± 0.6

## Data Availability

Data are contained within the article.
